# Cerebrospinal Fluid Volume and Other Intracranial Volumes Are Associated with Fazekas Score in Adults: A Single Center Experience

**DOI:** 10.3390/medicina61081411

**Published:** 2025-08-04

**Authors:** Melike Elif Kalfaoglu, Zeliha Cosgun, Aysenur Buz Yasar, Abdullah Emre Sarioglu, Gulali Aktas

**Affiliations:** 1 Department of Radiology, Bolu Abant Izzet Baysal University Hospital, 14020 Bolu, Turkey; melikekalfaoglu@hotmail.com (M.E.K.); zeliha44@gmail.com (Z.C.); aysenurbuz@gmail.com (A.B.Y.); a.emre.sarioglu@gmail.com (A.E.S.); 2 Department of Internal Medicine, Bolu Abant Izzet Baysal University Hospital, 14020 Bolu, Turkey

**Keywords:** cerebrospinal fluid, Fazekas score, volBrain

## Abstract

*Background and Objectives*: The objective of this research is to make a comparative evaluation of the correlation between the volumetric examination of subcortical cerebral regions and white matter hyperintensities classified according to the Fazekas scoring system. *Materials and Methods*: A total of 236 cases with cranial MRI studies were retrospectively analyzed. This study included patients aged over 45 years who had white matter hyperintensities and who did not have a prior stroke diagnosis. White matter hyperintensities were evaluated in axial FLAIR images according to Fazekas’s grading scale. Patients with Fazekas 0 and 1 were grouped in group 1 and the patients with Fazekas 2 and 3 were grouped in group 2. MRI data processing and subcortical volumetric analyses were performed using the volBrain MRI brain volumetry system. *Results*: There were statistically significant differences between groups 1 and 2 in terms of cerebrospinal fluid total brain white and gray matter (*p* < 0.001), total brain white and gray matter (*p* = 0.009), total cerebrum (*p* < 0.001), accumbens (*p* < 0.001), thalamus (*p* < 0.001), frontal lobe (*p* < 0.001), parietal lobe (*p* < 0.001), and lateral ventricle (*p* < 0.001) volumes. *Conclusions*: Our study finds a strong link between white matter hyperintensity burden and brain atrophy. This includes volume reductions in total brain white and gray matter, frontal and parietal lobe atrophy, increased cerebrospinal fluid (CSF), and atrophy in specific brain regions such as the accumbens and thalamus.

## 1. Introduction

In the later stages of life, structural alterations in the brain due to aging become pivotal factors influencing an individual’s health, behavior, and cognitive abilities. Aside from the well-documented gray matter atrophy, it is worth noting that elderly individuals often exhibit white matter changes, which can significantly impact cognitive function. White matter hyperintensities represent the primary expression of these white matter alterations [1]. White matter hyperintensities are commonly encountered as incidental findings in brain magnetic resonance imaging scans [2]. The histological composition of white matter hyperintensity is varied, involving both nonvascular and chronic ischemic alterations. Smooth periventricular and punctate subcortical and/or deep white matter hyperintensities can be attributed to nonvascular factors, indicating disturbances in ependymal structures accompanied by widened subependymal extracellular spaces and perivascular spaces, respectively. Conversely, confluent white matter lesions suggest ischemic modifications [3]. White matter hyperintensity is commonly associated with various factors, including hypertension, cerebrovascular disease (problems with blood vessels in the brain), and the normal aging process [4]. While the precise etiology remains incompletely understood, small vessel disease is widely recognized as the principal mechanism underlying age-related white matter hyperintensity. This pathology is characterized by diminished myelination, gliosis, and axonal damage, primarily arising from thickened vessel walls and luminal narrowing [5]. Various vascular risk factors, including hypertension, diabetes mellitus, hypercholesterolemia, smoking, and elevated homocysteine levels, have been identified as critical contributors to white matter hyperintensity development [6]. Furthermore, the prevalence of white matter hyperintensity exhibits an exponential increase with advancing age, affecting approximately 11–21% of individuals in their mid-60s in the general population and exceeding 90% in healthy, elderly individuals aged over 80 years. Importantly, white matter hyperintensities exhibit considerable heterogeneity in terms of both severity and anatomical distribution, ranging from minor punctate lesions to extensive damage in the deep and periventricular white matter regions. Research findings have pointed out that individuals displaying a 5-year advancement of continuous white matter lesions encounter heightened rates of cognitive deterioration [7].

White matter hyperintensities are areas of increased signal intensity on T2-weighted magnetic resonance imaging scans that appear as bright spots in the deep and subcortical white matter of the brain. The Fazekas scoring system is indeed a widely used method to assess the severity of white matter hyperintensities [3]. It helps radiologists and clinicians categorize and grade the extent of white matter hyperintensities seen on MRI scans [8]. The Fazekas scale typically ranges from 0 to 3: Fazekas Score 0: No white matter hyperintensities observed. Fazekas Score 1: Small punctate hyperintensities scattered in the white matter. Fazekas Score 2: More numerous and larger areas of hyperintensity, possibly connecting or merging. Fazekas Score 3: Large confluent areas of hyperintensity, possibly with involvement of the outermost layer of the brain (the cortex). The clinical significance of an elevated burden of cerebrovascular pathology is evident in the context of diagnosing individuals who possess an elevated risk for cerebrovascular disease [9]. Correspondingly, within clinical scenarios, a substantial predictive capacity is observed wherein elevated Fazekas WMSA burden accurately anticipates cognitive performance in patients afflicted with Alzheimer’s disease [10]. The Fazekas scoring system provides a simple and standardized way to communicate the severity of white matter hyperintensities, which can be valuable in clinical practice and research to track changes over time, evaluate disease progression, and guide treatment decisions. The association between white matter lesions and pathological changes is clearly evident. Utilizing volumetric measurements to quantify the loads of white matter hyperintensities can lead to improved diagnostic accuracy when evaluating neurological disorders. The magnitude of white matter hyperintensity burden offers insights into the holistic health of the brain, and there is a positive correlation between confluent white matter lesions and progressive brain atrophy [11].

Recently, various methodologies have been under scrutiny for the automatic or semi-automatic segmentation of subcortical structures. These include software tools tailored for the analysis and visualization of functional magnetic resonance neuroimages [12]; applications like BrainVoyager [13,14], FreeSurfer [15], and Mristudio [16,17]; and statistical parametric maps [13]. These software solutions are designed to elaborate upon the structural attributes of the human brain. In this context, the automated approach of volBrain stands out, where an observer can execute entirely automatic segmentation through a web-based application. Notably, volBrain was recently employed in a neuroimaging investigation involving MRI data [14].

In the context of the existing literature, the aim of this study is to comparatively assess the relationship between the volumetric analysis of subcortical brain structures and Fazekas score-classified white matter hyperintensities.

## 2. Materials and Methods

A retrospective, cross-sectional study was conducted in the Radiology Department of Abant Izzet Baysal University Hospital after obtaining ethical approval (Abant Izzet Baysal University Ethics Committee, date: 23 May 2023, approval no: 2023/172). Legal consent for the procedure was obtained from each patient.

The demographic information (age, gender, comorbidities, etc.) and recorded cranial magnetic resonance images (MRIs) of patients who visited our hospital between 1 August 2020 and 31 March 2023 were retrospectively retrieved from our hospital’s PACS system. This study included patients aged over 45 years who had white matter hyperintensities and who did not have a prior stroke diagnosis. Subcortical and periventricular white matter were evaluated in axial FLAIR images according to Fazekas’s grading scale. Two groups were formed: patients with Fazekas 0 and Fazekas 1 were grouped in group 1; patients with Fazekas 2 and Fazekas 3 were grouped in group 2. Participants with incomplete or insufficient data, recent significant trauma or surgery, a history of stroke, congenital central nervous system disorders, vasculitis, multiple sclerosis, intracranial masses, cancer, and conditions associated with elevated intracranial pressure were excluded from this study (*n* = 58). After applying the exclusion criteria, a total of 236 cases were included in this study.

We used a magnetic resonance imaging 1.5 T unit (Signa Explorer; GE Healthcare, Chicago, IL, USA, 2020) with a 19-channel head coil. The slice thickness was 5 mm. We obtained T1-weighted images (repetition time (TR)/echo time (TE)  = 2284/22.4 ms), T2-weighted images (TR/TE = 5710/105.6 ms), and T2 fluid-attenuated inversion recovery (FLAIR) images (TR/TE = 8000/91 ms). All MRI images were evaluated by two radiologists with 19 and 22 years of experience, and the Fazekas scores were determined by their joint decision, respectively. Evaluation of subcortical and periventricular white matter was conducted using the Fazekas grading scale on axial FLAIR images. Within the Fazekas grading scale, a rating of 1 was assigned to multiple dot-shaped lesions, a rating of 2 to confluent lesions, and a rating of 3 to large confluent lesions. Based on this grading system, the study cohort was divided into two categories: subjects with Fazekas 0 and Fazekas 1 constituted group 1 and subjects with Fazekas 2 and Fazekas 3 constituted group 2. The volumetric assessment was conducted on sagittal oblique T1-weighted images obtained perpendicular to the long axis of the subcortical structures. A BRAVO sequence was used with the following parameters: repetition time = 7.6 ms, echo time = 3 ms, field of view = 250 mm, slice thickness = 1 mm, voxel size = 1 × 1 × 1 mm.

**Figure 1:**
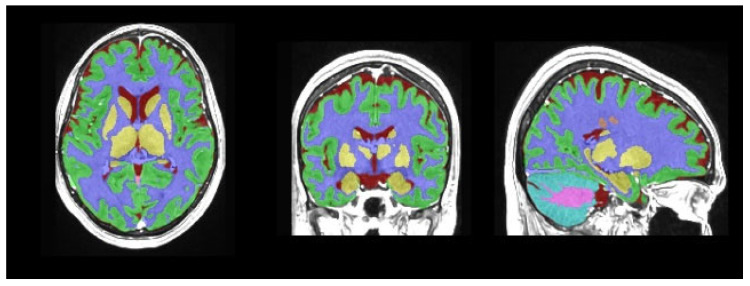
Tissue segmentation.

**Figure 2:**
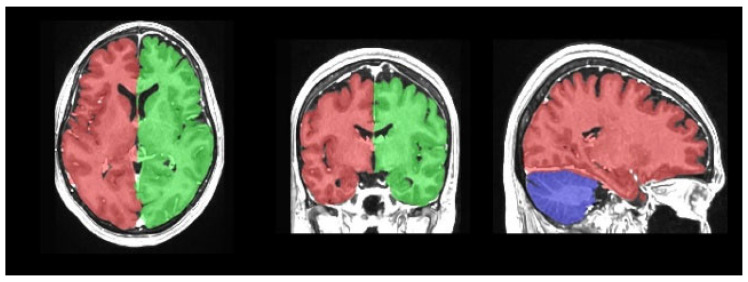
Macrostructures.

**Figure 3:**
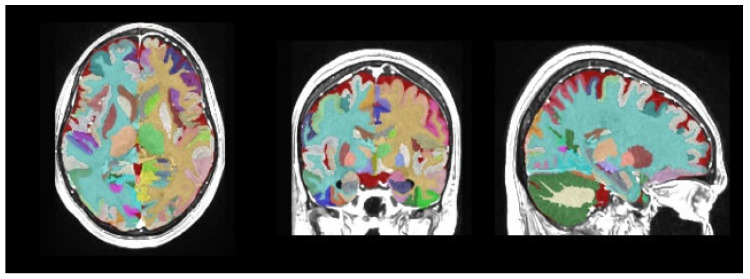
Structure segmentation.

Magnetic resonance imaging data processing and subcortical volumetric analyses were performed using volBrain (version 1.0, released 23 November 2021, http://volbrain.net (accessed 10 December 2024)), a free online MRI brain volumetry system. volBrain is a fully automated segmentation technique for which the algorithm is based on multi-atlas patch-based label fusion segmentation technology (Figure 1, Figure 2 and Figure 3).

volBrain is software that provides researchers with automated quantitative analysis of MRI data. It is capable of outlining structures and providing expected volumes for each considered structure according to the age and sex of the subjects under study. This capability could aid in detecting volumetric abnormalities and potential pathologies. volBrain is a pipeline dedicated to the automatic analysis of MRI brain data. It generates a report with the volumes of the main intracranial cavity (ICC) tissues (i.e., cerebrospinal fluid, gray matter, and white matter). Additionally, it offers volume information for certain macroscopic areas such as brain lobes, the cerebrum, and the brainstem. Finally, the software performs automatic subcortical structure segmentation, providing related volumes and label maps.

### Statistical Analyses

Statistical software (SPSS 18 for Windows, IBM Co., Chicago, IL, USA) was used for statistical analyses. The Kolmogorov–Smirnov test was applied to the study variables for normality analysis. Variables that fit into normal distribution were analyzed using an independent sample *t* test and results were expressed as means and standard deviations. Other variables that did not fit into normal distribution were expressed as medians (min–max) and compared using the Mann–Whitney U test. Categorical variables were compared between study groups with the chi-square test and given as numbers and percentages. Correlations between study variables were analyzed using Pearson’s correlation test. Specificity and sensitivity of study parameters in detecting Fazekas 2 and 3 were analyzed using the ROC analysis test. To find out whether study variables were independent risk factors for Fazekas progression, a logistic regression analysis was conducted, taking into account gender and volumes of CSF, brain white–gray matter, cerebrum, accumbens, thalamus, and frontal, parietal, and lateral ventricles. Results were considered significant when the *p* value was lower than 5%.

## 3. Results

A total of 236 subjects were enrolled in the present work after the exclusion of subjects according to the exclusion criteria. Of those, 114 were in group 1 and 122 were in group 2. The mean ages of groups 1 and 2 were 59 (37–81) years and 69 (34–95) years, respectively (*p* < 0.001). There were 72 (63%) women in group 1 and 60 (49%) women in group 2 (*p* = 0.03). Table 1 shows the demographic characteristics of the study population.

The mean total intracranial cavity (TIC) volumes of group 1 and group 2 were 1305 ± 122 mm^3^ and 1312 ± 137 mm^3^, respectively. There were no statistically significant differences between the study groups in terms of total intracranial cavity (TIC) volume (*p* = 0.37). The mean cortical gray matter volumes of group 1 and group 2 were 521 ± 59 mm^3^ and 510 ± 62 mm^3^, respectively (*p* = 0.68). The median subcortical gray matter volumes of group 1 and group 2 were 6.2 (4.6–9.8) mm^3^ and 6.1 (1.6–9.1) mm^3^, respectively (*p* = 0.48). The median cerebrum gray matter volumes of group 1 and group 2 were 558 (414–1314) mm^3^ and 542 (239–989) mm^3^, respectively (*p* = 0.11). The median caudate volumes of group 1 and group 2 were 38 (29–86) mm^3^ and 38 (22–49) mm^3^, respectively (*p* = 0.20). The median amygdala volumes of group 1 and group 2 were 1.9 (0.7–4.7) mm^3^ and 1.8 (1–9.1) mm^3^, respectively (*p* = 0.52). The mean globus pallidus volumes of group 1 and group 2 were 2.6 ± 0.4 mm^3^ and 2.6 ± 0.4 mm^3^, respectively (*p* = 0.51). The median putamen volumes of group 1 and group 2 were 7.3 (5.2–9.6) mm^3^ and 7.1 (1–9.8) mm^3^, respectively (*p* = 0.12). The median hippocampus volumes of group 1 and group 2 were 7.7 (2.8–9.9) mm^3^ and 7.8 (3.4–10.1) mm^3^, respectively (*p* = 0.53).

The mean temporal lobe volumes of group 1 and group 2 were 110 ± 12 mm^3^ and 107 ± 13 mm^3^, respectively (*p* = 0.78). The median occipital lobe volumes of group 1 and group 2 were 72 (46–95) mm^3^ and 72 (28–88) mm^3^, respectively (*p* = 0.88).

The median cerebrospinal fluid (CSF) volumes of groups 1 and 2 were 119 (53–292) mm^3^ and 193 (53–981) mm^3^, respectively. There were statistically significant differences between the study groups in terms of CSF volume (*p* < 0.001). The median total brain white and gray matter volumes in group 1 and group 2 were 1158 (825–1207) mm^3^ and 1113 (611–1491) mm^3^, respectively. Statistically significant differences were observed between the study groups regarding the combined volume of total brain white and gray matter (*p* = 0.009). The median total cerebrum volumes in group 1 and group 2 were 1046 (734–1257) mm^3^ and 992 (492–14,326) mm^3^, respectively. There were statistically significant differences between the study groups in terms of total cerebrum volume (*p* < 0.001). The median accumbens volumes of groups 1 and 2 were 0.6 (0.3–0.9) mm^3^ and 0.5 (0.2–0.9) mm^3^, respectively. A statistically significant difference in accumbens volume was observed between the study groups (*p* < 0.001). The median thalamus volumes of groups 1 and 2 were 11.8 (8.1–16.2) mm^3^ and 10.8 (3.3–16.4) mm^3^, respectively. There was a statistically significant difference between the study groups in terms of thalamus volume (*p* < 0.001). The median frontal lobe volumes of groups 1 and 2 were 180 (124–255) mm^3^ and 167 (53–250) mm^3^, respectively. There was a statistically significant difference between the study groups in terms of thalamus volume (*p* < 0.001). The median parietal lobe volumes of groups 1 and 2 were 85 (69–132) mm^3^ and 96 (43–143) mm^3^, respectively. There was a statistically significant difference between the study groups in terms of parietal lobe volume (*p* < 0.001). The median lateral ventricle volumes of groups 1 and 2 were 22 (7–87) mm^3^ and 33 (8–95) mm^3^, respectively. A statistically significant difference in lateral ventricle volume was observed between the study groups (*p* < 0.001). The median cerebrum white matter volumes in group 1 and group 2 were 461 (232–688) mm^3^ and 441 (134–889) mm^3^, respectively. There was a statistically significant difference between the study groups in cerebrum white matter volume (*p* = 0.049). Table 2 shows the data for the study groups.

In the correlation analysis, CSF volume was positively and significantly correlated with lateral ventricle volume (r = 0.22, *p* = 0.001). CSF volume was negatively and significantly correlated with accumbens (r = −0.15, *p* = 0.02), frontal lobe (r = −0.20, *p* = 0.002), and thalamus (r = −0.33, *p* < 0.001) volumes. LV volume was negatively and significantly correlated with cerebrum total (r = −0.14, *p* = 0.03), cerebrum white matter (r = −0.2, *p* < 0.001), accumbens (r = −0.31, *p* < 0.001), and thalamus (r = −0.24, *p* < 0.001) volumes. Correlations between study variables are presented in Table 3.

**Figure 4:**
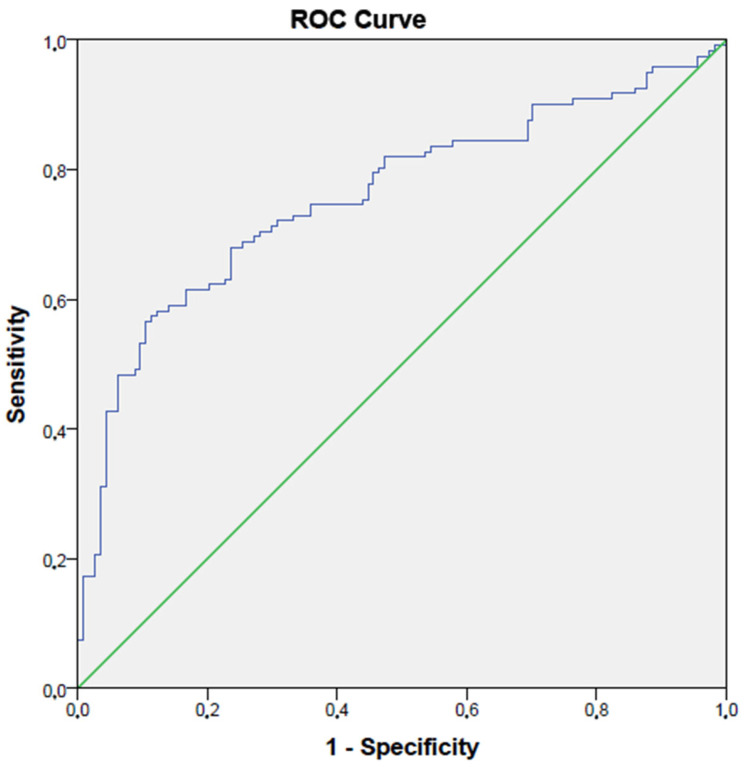
The ROC curve of CSF in detecting Fazekas groups.

In the ROC analysis, a CSF volume higher than 145 had 69% sensitivity and 73% specificity in detecting group 2 (AUC: 0.76, *p* < 0.001, 95% CI: 0.70–0.82) (Figure 4).

## 4. Discussion

Brain tissue atrophy represents a widespread phenomenon associated with the process of getting older [15]. Nevertheless, recent research involving machine learning techniques showed that the aging of the brain is a phenomenon that can be separated from one’s actual chronological age. This means that the brain can either “age slower or faster” than what would be expected based purely on the number of years a person has lived [16]. The exact reasons behind this decoupling of chronological age from brain aging are not entirely clear, but they may be related to the distinct physiological and dynamic characteristics of the central nervous system, with brain plasticity potentially acting as a protective mechanism [17]. Conversely, premature brain aging could result from a particular vulnerability to chronic damage due to the brain’s high metabolic demands, especially in cases of chronic insufficient blood supply to the brain. To date, researchers have established strong connections between age and overall structural alterations in the brain, including changes in white matter, gray matter, and cerebrospinal fluid [18]. To better understand the mechanisms associated with premature brain aging, it is essential to consider white matter hyperintensities (WMHs). WMHs are markers observed in neuroimaging that indicate the presence of small vessel disease, which can be linked to chronic inadequate blood supply to the brain [19]. These markers were previously considered innocuous findings but may actually signify structural changes in the brain, with a subtle impact on cognitive abilities, particularly in terms of executive function [20,21]. Even in younger individuals with a low burden of WMHs, a connection between WMH load and working memory has been observed. The association between chronological age and the extent of WMHs is well-documented, with older individuals generally exhibiting a greater load of WMHs [22]. Due to this association, recent research has also attempted to predict a person’s chronological age based on measurements of WMH load [23]. However, these studies have typically focused on the link between WMH load and chronological age rather than the age of the brain. It is worth noting that assessments of cortical integrity using measurements of brain age generally rely on structural T1-weighted MRI scans, while other imaging techniques, such as T2-weighted or T2-FLAIR scans, are better suited for quantifying age-related WMH load [24]. The need for different imaging modalities is one of the reasons why research has primarily concentrated on the relationship between WMH load and chronological age, rather than brain age.

In our study, we conducted visual classification using the Fazekas scale and accompanying volumetric measurements for white matter hyperintensities. A significant difference, consistent with the literature, was observed between the volumetric measurement of white matter hyperintensities and the Fazekas classification [25,26]. For Fazekas grade 1, the average white matter hyperintensity burden was 7.3, whereas it was calculated as 254.9 for Fazekas grade 3. This demonstrates the concordance between classification based on visual assessment and volumetric measurement [8]. However, the presence of numerical data in volumetric analysis enhances the value of this method in the evaluation of white matter hyperintensities. The Fazekas classification system provides us with a practical method for evaluating white matter lesions. However, in patient groups where tracking the burden of hyperintensities is necessary, it would be more appropriate to evaluate small differences in hyperintensity burden using volumetric measurements in conjunction with the Fazekas classification [27,28]. In patients with multiple sclerosis, multiple white matter hyperintensities have been observed, and the emergence of new lesions during follow-up is crucial for guiding treatment. Therefore, there are studies recommending volumetric analysis in the monitoring of multiple sclerosis patients [29].

The second important finding of our study is the significant volume difference between the thalamus and accumbens among the Fazekas groups. In our study, there was a significant age difference among the Fazekas groups, with an average age of approximately 59 in Fazekas 1 and 72 in Fazekas 3. Volumetric studies focusing on the brain have found that the accumbens and thalamus are regions that are more affected by advancing age compared to other anatomical structures, and their volumes decrease with age [30]. In our study, consistent with the literature, we observed a decrease in accumbens and thalamus volumes with advancing age and concomitant increased white matter hyperintensity burden. The relationship between subcortical atrophy and the Fazekas classification, considering the association of white matter lesions with increasing age, neurocognitive impairments, and dementia, was evaluated in line with the literature [31].

Another significant finding of our study was the relationship between white matter hyperintensity burden and hippocampal volume loss. In our study, reduced hippocampal volume was observed in the Fazekas 3 group compared to the Fazekas 1 group. The hippocampus is a brain region sensitive to global hypoperfusion, and pathological studies have shown a series of events triggered by impaired microvascular perfusion leading to gray matter atrophy. Despite the small sample size, the statistically significant relationship between hippocampal volume loss and white matter hyperintensity burden in our study is in line with the existing literature, thus supporting it [32,33].

White matter hyperintensities are linked to a higher risk of cognitive decline. In a comprehensive study involving a substantial population sample, Habes and colleagues demonstrated that white matter hyperintensity burden plays a role in the atrophy of brain regions typically affected by accelerated brain aging and Alzheimer’s disease [34]. Additionally, previous research has shown that a high burden of white matter hyperintensities is associated with both total gray matter atrophy [35,36] and atrophy in specific brain regions such as the temporal lobe and frontal cortex [37,38]. Our study found a significant relationship between white matter hyperintensity burden and brain atrophy, which was consistent with the literature. Similar to the study conducted by Aribisala et al., we examined variables such as cerebrospinal fluid volume, lateral ventricle volume, and total brain volume. An increase in cerebrospinal fluid volume and lateral ventricle volume, in line with our results, was correlated with a decrease in total brain volume, indicative of white matter hyperintensity burden. Furthermore, akin to the research carried out by Tuladhar et al., we evaluated the association between white matter hyperintensity burden and cortical and subcortical gray matter. Volumetric measurements of cortical and subcortical gray matter correlated with white matter hyperintensity burden. The relationship between increased white matter hyperintensity burden and observed volume loss in cortical and subcortical gray matter is statistically significant. White matter hyperintensities have been linked to cognitive impairment, increased stroke risk, and dementia in numerous studies [39]. The presence of accompanying cortical atrophy elevates these patients to a higher-risk group for dementia [40]. Therefore, we believe that identifying concomitant cortical atrophy through volumetric measurements in patients with Fazekas 3 lesions would be beneficial in predicting high-risk groups for dementia.

In our study, we conducted separate volumetric measurements and analyses for the frontal, temporal, parietal, and occipital lobes, and we found a significant relationship between white matter hyperintensity burden and volume loss only in the temporal lobe. While medial temporal lobe atrophy is frequently mentioned in the literature; our study did not specifically investigate the volume of the medial temporal lobe [41].

The present study showed that cerebrospinal fluid volume was an independent risk factor for increased Fazekas scores in adult subjects when adjusted for brain white matter–gray matter volume, cereberum total volume, accumbens volume, thalamus volume, frontal lobe volume, and lateral ventricle volume. This finding suggests that CSF volume is independently associated with a higher Fazekas score, which reflects the severity of white matter hyperintensities (WMHs), even after adjusting for multiple brain volumetric measures, including gray matter (GM), white matter (WM), total cerebrum, accumbens, thalamus, frontal lobe, and lateral ventricle volumes. Several studies have identified that increased CSF volume (reflecting brain atrophy and ventricular enlargement) correlates with the burden of WMHs. This relationship is thought to be due to several reasons. One of them is perivascular clearance dysfunction. Impaired CSF dynamics may hinder the removal of interstitial waste and promote WM injury. Another possible cause could be brain atrophy. As brain tissue shrinks (normal aging, neurodegeneration), CSF spaces enlarge; however, our finding suggests that CSF volume itself, independent of tissue atrophy, predicts WMHs. This association remained significant even after adjusting for multiple volumetric brain parameters, including white matter (WM) volume, gray matter (GM) volume, total cerebrum volume, nucleus accumbens volume, thalamus volume, frontal and parietal lobe volumes, and lateral ventricle volume. Our findings are consistent with accumulating evidence highlighting the role of CSF dynamics in the pathogenesis of cerebral small vessel disease (SVD) and WMHs. Previous studies have shown that enlarged CSF spaces, including the ventricles and subarachnoid space, are associated with an increased WMH burden [42]. Importantly, several studies emphasize that CSF space enlargement may reflect not only brain atrophy but also impaired interstitial fluid clearance and glymphatic dysfunction, which are increasingly recognized as contributors to WM injury [43,44]. The significance of CSF volume as an independent predictor suggests a potential mechanistic link beyond simple brain atrophy. Enlarged CSF spaces may reflect impaired glymphatic clearance or disruptions in CSF–interstitial fluid exchange, leading to the accumulation of neurotoxic metabolites and promoting white matter damage [45]. Additionally, mechanical effects due to increased CSF pressure or periventricular shear stress may contribute to the development of periventricular WMHs, as supported by imaging studies demonstrating close proximity between enlarged ventricles and WMH distribution [46]. Furthermore, our finding aligns with prior observations demonstrating that perivascular spaces and ventricular enlargement are closely associated with WMH burden, independent of age and brain atrophy [47]. This supports the hypothesis that altered CSF dynamics may be a modifiable risk factor for cerebral microvascular disease, potentially offering new avenues for prevention or treatment. From a clinical perspective, our results suggest that CSF volume measurements could serve as an early imaging biomarker to identify individuals at a higher risk of WMH progression, even in the absence of overt brain tissue loss. Given the growing recognition of WMHs as predictors of cognitive decline, gait impairment, and increased stroke risk, the early detection of CSF-related changes may have important prognostic and therapeutic implications.

The most significant limitation in our study is the limited number of patients. This was because this study was retrospectively planned and detailed clinical data were not available.

In this article, an automated and reliable analysis was employed to investigate volumetric changes [14]. A novel method using volBrain was introduced, demonstrating its potential as a substitute for conventional volumetric techniques. volBrain offers several advantages for brain imaging researchers, notably providing swift results with minimal fatigue in the context of treatments and clinical studies related to neurological disorders. One primary limitation of our study is its non-longitudinal nature. To corroborate the findings presented herein, a larger participant pool and further studies are required. Another constraint is the absence of manual volumetric measurements. Notably, volBrain is versatile and can be applied to measure volumes of various anatomical regions throughout the body using radiological images. It is our belief that the outcomes of this study will augment the existing knowledge in volumetric investigations concerning the development, pathology, and anomalies of subcortical structures.

## 5. Conclusions

In conclusion, our study demonstrated a significant association between white matter hyperintensity burden and total brain atrophy, volume loss in cortical and subcortical gray matter, temporal lobe atrophy, and volume loss in specific brain structures such as the subcortical accumbens, thalamus, and hippocampus. The findings of the present work suggest that volumetric analysis may reveal subtle atrophy in brain structures that is not visually apparent, thereby enhancing diagnostic accuracy in patients with increased white matter hyperintensities.

## Figures and Tables

**Table 1:** Demographic characteristics of the study population.

		Group 1 (*n* = 114)	Group 2 (*n* = 122)	*p*
Gender (*n*, %)	Women	72 (63%)	60 (49%)	<0.001
	Men	42 (37%)	62 (51%)	<0.001
Age (years)		59 (37–81)	69 (34–95)	<0.001

**Table 2:** Data of the study population.

Volume (mm^3^)	Group 1 (*n* = 114)	Group 2 (*n* = 122)	*p*
	Mean ± Std		
Total intracranial cavity	1305 ± 122	1312 ± 137	0.37
Cortical gray matter	521 ± 59	510 ± 62	0.68
Globus pallidus	2.6 ± 0.4	2.6 ± 0.4	0.51
Temporal lobe	110 ± 12	107 ± 13	0.78
	Median (Min–Max)		
Subcortical gray matter	6.2 (4.6–9.8)	6.1 (1.6–9.1)	0.48
Cerebrospinal fluid	119 (53–292)	193 (53–981)	<0.001
Total brain white + gray matter	1158 (825–1207)	1113 (611–1491)	0.009
Total cerebrum	1046 (734–1257)	992 (492–1326)	<0.001
Cerebrum white matter	461 (232–688)	441 (134–889)	0.049
Cerebrum gray matter	558 (414–1314)	542 (239–989)	0.11
Accumbens	0.6 (0.3–0.9)	0.5 (0.2–0.9)	<0.001
Amygdala	1.9 (0.7–4.7)	1.8 (1–9.1)	0.52
Caudate	38 (29–86)	38 (22–49)	0.20
Putamen	7.3 (5.2–9.6)	7.1 (1–9.8)	0.12
Thalamus	11.8 (8.1–16.2)	10.8 (3.3–16.4)	<0.001
Hippocampus	7.7 (2.8–9.9)	7.8 (3.4–10.1)	0.53
Frontal lobe	180 (124–255)	167 (53–250)	<0.001
Occipital lobe	72 (46–95)	72 (28–88)	0.88
Parietal lobe	85 (69–132)	96 (43–143)	<0.001
Lateral ventricle	22 (7–87)	33 (8–95)	<0.001

**Table 3:** Correlations between study variables.

	CSF Volume	Total Brain White + Gray Matter	Total Cerebrum	Cerebrum White Matter Volume	Accumbens Volume	Thalamus Volume	Frontal Lobe Volume	Parietal Lobe Volume	Lateral Ventricle Volume
CSF volume	-	NS	NS	NS	r = −015 *p* = 0.02	r = −0.33 *p* < 0.001	r = −0.20 *p* = 0.02	r = 0.13 *p* = 0.04	r = 0.22 *p* < 0.001
Total brain white + gray matter	NS	-	r = 0.20 *p* = 0.003	r = 0.15 *p* = 0.025	NS	NS	r = 0.14 *p* = 0.033	NS	NS
Total cerebrum	NS	r = 0.20 *p* = 0.003	-	r = 0.68 *p* < 0.001	r = 0.61 *p* < 0.001	r = 0.66 *p* < 0.001	r = 0.78 *p* < 0.001	r = 0.48 *p* < 0.001	r = −0.14 *p* = 0.029
Cerebrum white matter volume	NS	r = 0.15 *p* = 0.025	r = 0.68 *p* < 0.001	-	r = 0.32 *p* < 0.001	r = 0.34 *p* < 0.001	r = 0.34 *p* < 0.001	r = 0.40 *p* < 0.001	r = −0.20 *p* = 0.002
Accumbens volume	NS	r = −015 *p* = 0.02	r = 0.61 *p* < 0.001	r = 0.32 *p* < 0.001	-	r = 0.61 *p* < 0.001	r = 0.60 *p* < 0.001	r = 0.32 *p* < 0.001	r = −0.31 *p* < 0.001
Thalamus volume	r = −0.33 *p* < 0.001	NS	r = 0.66 *p* < 0.001	r = 0.34 *p* < 0.001	r = 0.61 *p* < 0.001	-	r = 0.65 *p* < 0.001	r = 0.25 *p* < 0.001	r = −0.24 *p* < 0.001
Frontal lobe volume	r = −0.20 *p* = 0.02	r = 0.14 *p* = 0.033	r = 0.78 *p* < 0.001	r = 0.34 *p* < 0.001	r = 0.60 *p* < 0.001	r = 0.65 *p* < 0.001	-	r = 0.37 *p* < 0.001	NS
Parietal lobe volume	r = 0.13 *p* = 0.04	NS	r = 0.48 *p* < 0.001	r = 0.40 *p* < 0.001	r = 0.32 *p* < 0.001	r = 0.25 *p* < 0.001	r = 0.37 *p* < 0.001	-	NS
Lateral ventricle volume	r = 0.22 *p* < 0.001	NS	r = −0.14 *p* = 0.029	r = −0.20 *p* = 0.002	r = −0.31 *p* < 0.001	r = −0.24 *p* < 0.001	NS	NS	-

## Data Availability

Data for this work is available upon request from the corresponding author.
